# Quasi-Monocrystalline Graphene Crystallization on Liquid Copper Matrix

**DOI:** 10.3390/ma13112606

**Published:** 2020-06-08

**Authors:** Dominika Kuten, Konrad Dybowski, Radomir Atraszkiewicz, Piotr Kula

**Affiliations:** 1 Advanced Graphene Products Sp. z o.o., Nowy Kisielin A. Wysockiego 4, 66-002 Zielona Góra, Poland; 2 Institute of Materials Science and Engineering, Lodz University of Technology, 1/15 Stefanowskiego, 90-924 Łódź, Poland; konrad.dybowski@p.lodz.pl (K.D.); radomir.atraszkiewicz@p.lodz.pl (R.A.); piotr.kula@p.lodz.pl (P.K.)

**Keywords:** crystal structure, graphene growth, low-angle boundaries, nanomaterials

## Abstract

To access the properties of theoretical graphene, it is crucial to manufacture layers with a defect-free structure. The imperfections of the structure are the cause of deterioration in both electrical and mechanical properties. Among the most commonly occurring crystalline defects, there are grain boundaries and overlapping zones. Hence, perfect graphene shall be monocrystalline, which is difficult and expensive to obtain. An alternative to monocrystalline structure is a quasi-monocrystalline graphene with low angle-type boundaries without the local overlapping of neighboring flakes. The purpose of this work was to identify factors that directly affect the structure of graphene grown on a surface of a liquid metal. In the article the growth of graphene on a liquid copper is presented. Nucleating graphene flakes are able to move with three degrees of freedom creating low-angle type boundaries when they attach to one another. The structure of graphene grown with the use of this method is almost free of overlapping zones. In addition, the article presents the influence of impurities on the amount of crystallization nuclei formed, and thus the possibility to order the structure, creating a quasi-monocrystalline layer.

**Highlights**

- Metallurgical graphene is grown on a liquid metal surface
- Graphene grain boundaries obtained in the research are of low-angle type
- The lower the nucleation rate, the more ordered the quasi-monocrystalline structure
- A large number of impurities prevents the formation of an ordered (quasi-monocrystalline) structure
- Impurities initiate heterogeneous nucleation of graphene nuclei

## 1. Introduction

Graphene is one of the carbon derivatives with sp2 hybridization. In graphene, carbon atoms are arranged in a honeycomb structure, which provides the material with its outstanding properties, such as chemical stability, optical transparency, enormous mechanical strength and electrical characteristics [1]. Graphene has a great potential for various applications such as high-quality composites for the automotive and aircraft industry, energy storage, filtration of liquids and anticorrosive protection. [1,2,3,4,5].

However, as a nanomaterial, especially in the powder form, graphene can cause a serious health hazard through pulmonary, oral or dermal exposure, where inhalation causes the greatest risk [6,7,8,9]. What is more, depending on the production method, graphene may strongly influence environmental safety (e.g., graphene oxide production requires strong, concentrated acids that generate hazardous waste which must be disposed of [10]). On the other hand, large-area graphene (which occurs in the form of sheets with dimensions from a few cm^2^ to even 1 m^2^ and bigger) is a much safer form of graphene, since it is highly unlikely to be inhaled. Processing large-area graphene, namely transferring it to desired substrates, requires the usage of metal etchants (wet transfer) or electrolyte solutions (hydrogen delamination) [11], that again, have to be disposed of.

For the commercial implementation of graphene, a scalable, repeatable and efficient production technique must be developed [12]. Production methods for graphene powder have been known for decades now. In the case of large-area graphene, chemical vapor deposition (CVD) or epitaxial growth from silicon carbide became widely used methods after the Nobel Prize was awarded for confirming the properties of graphene [1,2,3,4,12]. Since CVD is a low-cost and highly scalable process [13] with a wide range of possible growth substrates (Cu, Ni, Pt, etc.), it is the leading method of graphene production [1,3,13]. The choice of the catalytic substrate is crucial to the decomposition of carbon sources. Moreover, it influences the growth behavior and the structure, including the number of layers, size distribution and nucleation density. The solubility of carbon in a substrate determines the carbon diffusion depth and growth mechanism [14]. The low solubility of carbon in copper restrains the formation of multilayers through the suppression of carbon segregation in metal [14]. The low reactivity with carbon is owed to the fact that copper has a filled 3d-electron shell, the most stable configuration due to symmetrical electron distribution which minimizes reciprocal repulsions [14]. As a consequence, copper can only form weak bonds with carbon [15]. However, the structure imperfections of the polycrystalline growth substrate, including numerous grain boundaries, are the first cause of graphene degradation [4,13]. The polycrystalline growth of graphene is favored due to several grain boundaries of the growth substrate—the domain nucleation tends to occur with different in-plane orientations. Graphene islands nucleated at copper’s crystalline mismatches and defects tend to be polycrystalline, while single graphene crystals nucleate on the flatter regions. Aside from the polycrystallinity of graphene film, every surface imperfection could be considered as an impurity with a high chemical activation energy. The impurity acts like an active site attracting more carbon atoms, causing the nonuniformity of the graphene layer and the local formation of a multilayer [16,17,18,19,20]. Hence, polycrystalline graphene grown on a polycrystalline substrate will show inferior properties in comparison with graphene grown on a monocrystal [21]. There are several approaches to solve this problem and produce large scale single graphene crystals. One is to decrease the number of nucleation seeds. However, this approach requires a meticulous surface preparation. Another approach is to decrease the number of polycrystals in the substrate, which can be achieved by surface electro-polishing, for instance, that removes crystal defects (active sites) [22,23,24]. However, the results obtained by Zhao et al. [25] prove that electro-polishing may not be enough. Graphene obtained on polished Cu surface has limited coverage and contains randomly oriented islands with respect to the substrate crystal surface. Moreover, it has worse microstructure quality in comparison with graphene grown on the monocrystalline surface.

Another approach is to slow down the nucleation rate, to achieve a limited number of graphene nuclei. That will allow the formation of larger single crystals before neighboring islands merge [17,26,27]. It was proved that the presence of oxygen within the surface of copper foil can initiate the growth of large single crystals. After the decomposition of the copper oxide layer in a non-reducing atmosphere, some trace amounts of oxygen atoms remain at the surface of the substrate, which causes the drastic decrease in graphene nucleation density. Still, surface roughness and defects affect the quality of graphene.

The graphene synthesis on liquid metal takes advantage of the quasi-atomically smooth surface of the liquid, which prevents the negative influence of defects and grain boundaries present in solids [4]. The graphene nucleation and growth mechanism in liquids—as well as the size, shape, quality and thickness control of such liquid—are expected to be different from solid substrates due to the thermally enhanced surface migration of atoms and different catalytic behaviors [3].

Liquid metals have demonstrated a great potential to synthesize large-area, uniform and almost defect-free graphene sheets. Liquid can play the role of a matrix for carbon deposition in a CVD process, or a solvent for carbon atoms to dissolve in, from which the graphene precipitates out further in the process in the process the graphene precipitates out [13]. Using a liquid growth surface can provide a high diffusion rate and the fast growth of nuclei. Liquid has the smoothest surface which additionally provides three degrees of freedom for emerging nuclei, allowing them to assemble along their edges. If the growth continues, neighboring graphene islands form an ordered and compact structure. Additionally, synthesis on liquid surfaces showed different shapes of graphene grains, including dendritic, round, snowflake-like, hexagonal, or even twelve-pointed single crystals [28,29,30,31].

Several liquid metal substrates have been reported suitable for graphene growth, including copper, gallium and silver [4,32,33,34,35,36,37,38,39]. Unfortunately, the majority of melted metals cannot spread on the surface of a supporting substrate at reduced temperatures and tend to remain in an energetically favorable spherical state. Spreading or staying in a sphere is strongly dependent on the surface tension which, in turn, is highly related to the strength of cohesive forces between neighboring molecules [22]. In order to produce large and uniform graphene film areas, the excellent spreading of a liquid matrix on a supporting substrate is a must. Melted copper has a high surface energy and cannot extend over common supporting substrates. Although molten Cu does wet some metal foils, e.g., W or Mo. [12], nickel has better wettability by copper than molybdenum and tungsten, because the Cu–Ni phase equilibrium system is characterized by unlimited solubility in both the solid and liquid state. Therefore, at the heating stage for copper melting, a good diffusion connection of these metals is formed, and after melting the copper, it spreads smoothly and evenly on the surface of the nickel.

In this report, an investigation of graphene growth on liquid copper via precipitation, and how this growth is affected by solid impurities, is presented. Liquid copper was chosen because the liquid is perfectly smooth at the sub-micrometer level with no surface defects, which is an issue in the case of solid substrates. Graphene grown on a solid metal substrate reproduces the surface defects of the growth substrate. In addition, the liquid surface allows the formed nuclei to move and rotate, which creates the possibility of creating an ordered structure, as demonstrated by the article. Another raised issue was the dependence of graphene nuclei mobility on their number and density. The aim of this research was to obtain a monolayer of graphene with low-angle type boundaries in which properties are closer to the theoretical ones, as it was described by Kula et al. [40].

## 2. Materials and Methods

Graphene was grown on a liquid copper surface inside a molybdenum crucible. As mentioned before, copper can spread only over several metals, among which nickel it the most suitable. Since it has better wettability than molybdenum, the nickel layer was deposited on the crucible. A nickel layer with a thickness of 0.2 µm was applied on a molybdenum crucible by the Radio Frequency Physical Vapor Deposition (RF PVD) method. Before putting on the nickel coating, the surface of the samples was subjected to argon plasma etching with a pressure of 2 Pa, auto-polarization potential of −800 V and a time of 10 min. Plasma etching was applied to remove any impurities from the crucible surface. Then, the coating was applied using DC pulse magnetron sputtering (self-constructed device, Lodz University of Technology, Łódź, Poland) with a power of 1.0 kW, a pressure of 0.5 Pa, a potential of −50 V and a time of 30 min. The dimensions of the crucible were 53 mm in diameter and 5 mm in height. Then, a copper disc (Henan Guoxi Ultrapure New Materials Co., Ltd, Pingdingshan, China) with a 52 mm diameter and a 1.0 mm thickness was put into the crucible. The copper purity was 99.9995%.

The graphene synthesis was conducted in a vacuum furnace (SuperCarb, Seco/Warwick, Świebodzin, Poland). After inserting the samples, the furnace was pumped to a pressure of 10 Pa. The next step of the process consisted of heating the substrate to the temperature of 1100 °C under argon with a 3% hydrogen atmosphere. The pressure was maintained at 10 kPa. The growth substrate was kept at these conditions for 10 min. Eventually, the system was cooled down to 1050 °C with a cooling rate of 0.5 °C/min in the same atmosphere. The source of carbon was the mixture of acetylene, ethylene and hydrogen in a mass flow proportion of 2:2:1, respectively. The gas mixture was simultaneously added into the reaction chamber for 10 min with a flow rate of 5 L/min during the initial thermal stage of 1100 °C. The synthesis was conducted according to the patent [41].

A Hitachi scanning electron microscope (S-3000M, Hitachi High Technologies, Tokio, Japan), working in secondary electron (SE) mode at 5 kV accelerating voltage, was used for the qualitative morphology analysis of the metallurgical graphene layers.

Raman spectroscopy (inVia Reflex, Renishaw, Wotton-under-Edge, UK) with an excited wavelength of 532 nm was used to characterize the graphene.

A graphene layer was separated from the growth substrate using the electrochemical method and transferred into a copper mesh. Quality measurements of the obtained graphene were conducted with a transmission electron microscope (TEM). For this purpose, an HR TEM ((Talos F200X FEI, Thermo Fisher Scientific, Waltham, MA, USA) was used with an atomic resolution microscope) with a maximum accelerating voltage of 200 kV.

## 3. Results and Discussion

The most common structural defects are graphene grain boundaries [21,42,43,44,45,46,47,48,49], which were found to degrade graphene properties, such as its electrical quality, thermal conductivity, mechanical strength or oxidation resistance [21,26,40,45,50,51,52,53,54]. Graphene nucleation at multiple sites and the formation of graphene domains causes the formation of grain boundaries. Alongside those boundaries, one or more crystal disconnections to the neighboring domain may occur [21,55], especially for high-angle grain boundaries. When the dislocation cores become crowded, the grain boundaries tend to overlap [56]. This can be the cause of discontinuity in a graphene layer, which leads to the oxidation of copper beneath it [50,57]. In most CVD processes, the formation of graphene flakes is a random mechanism with no control over their orientation and placement. While the connection between the atomically stitched grain boundaries was due to covalent bonding, the overlap regions formed local bi-layers held together by van der Waals forces. The overlapping leads to a further decrease in the mechanical and thermal properties. Even up to 40% of grain boundaries could be overlapped with irregular misorientation [42,43,57,58,59]. Overlapping takes place during the fusion of two domains with different crystalline orientation varying between 10° and 25° [42].

**Figure 1:**
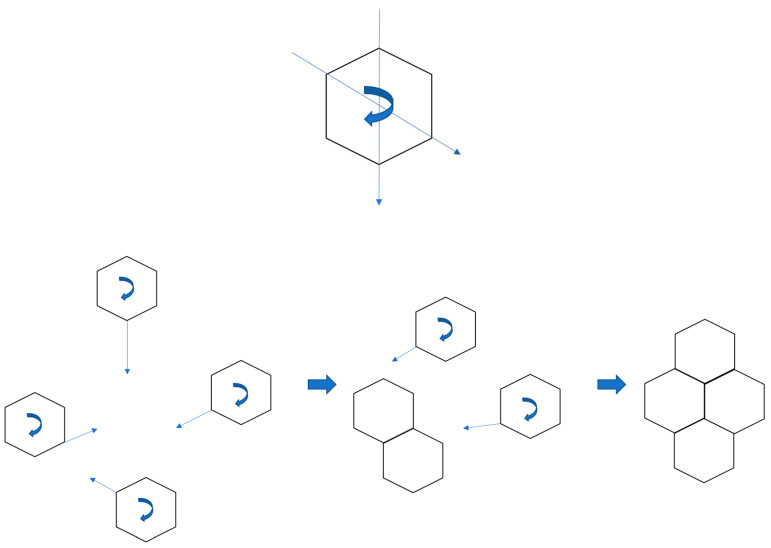
Schematic representation of a mechanism of the self-organizing formation of a graphene layer.

By measuring the misorientation angles, we proved that in the case of high-strength metallurgical graphene, there are low-angle grain boundaries at the misorientation angle below 10°. This phenomenon was a consequence of the growth mechanism on a liquid phase. Graphene islands appearing on a liquid surface have three degrees of freedom: they are able to move and rotate in the XY plane, which is shown in Figure 1. It is impossible in the case of growth on a solid substrate. As was mentioned above, the nucleation of graphene grains is determined by the crystallographic structure and topography of the substrate surface. The solid surface always has some roughness and irregularity, which leads to the nonuniform crystallinity of the graphene layer and the overlapping of grain boundaries. The monocrystalline graphene can be achieved only by growth on a monocrystal, which is a rather expensive material; or on a liquid surface, which is considerably more economical than monocrystals.

**Figure 2:**
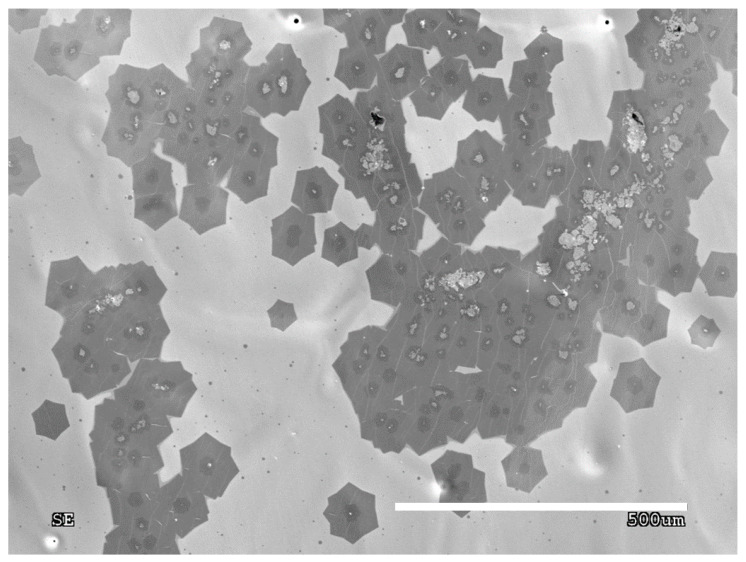
SEM images of the graphene nucleation and its impurities.

**Figure 3:**
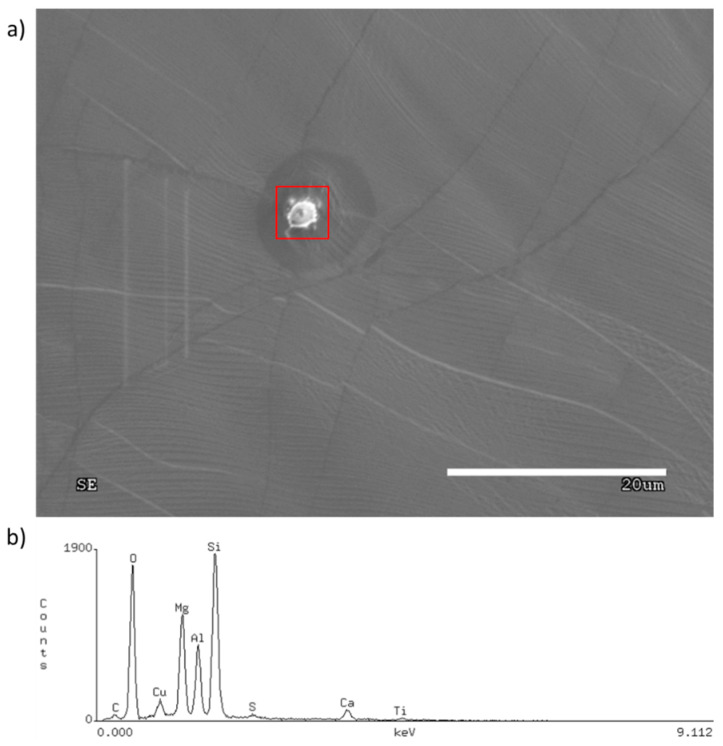
(**a**) SEM image of the foreign particle; and (**b**) EDS spectrum of a foreign particle.

The process purity is one of the main factors which determines the number of appearing nuclei and their sizes. Each foreign particle on the growth surface acts as an active site, favoring the heterogenous graphene nucleation starting with those particles. It is clearly visible that the particle is in the center of the forming graphene flakes. The same impurity particle can be an active site for another graphene nuclei. Figure 2 presents the scanning electron microscopy (SEM) image of graphene flakes with foreign particles inside them. The impurities were examined using energy dispersive spectroscopy (EDS), which is shown in Figure 3.

**Figure 4:**
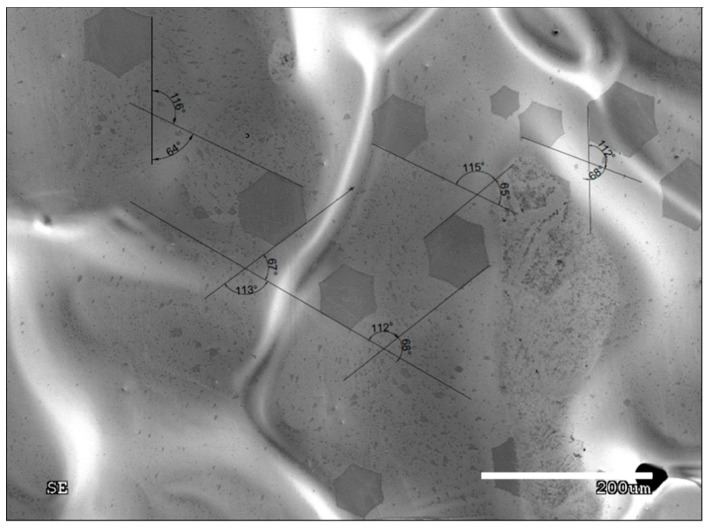
SEM image of the misorientation angles between the individual graphene domains.

**Figure 5:**
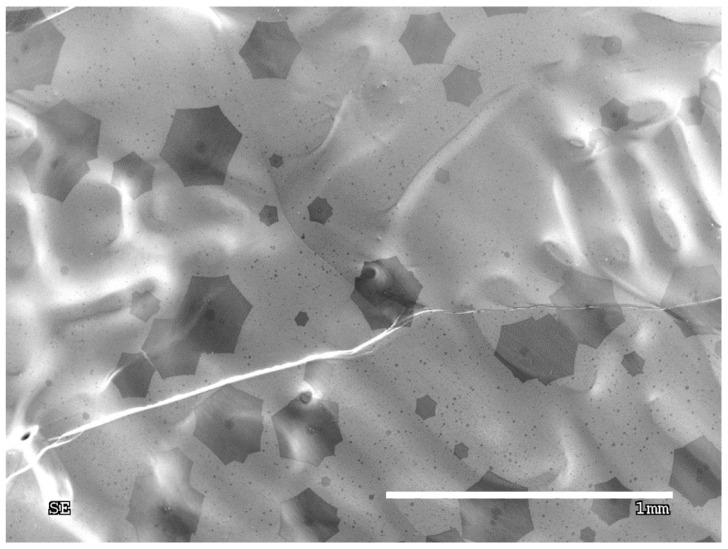
SEM image of a reduced number of nuclei, which are able to rotate and rearrange until fitting perfectly to each other.

Based on the EDS spectrum (Figure 3), the particle was identified as ceramics, most probably derived from the ceramic furnace components. As mentioned previously, these particles behave like active sites, therefore, graphene nuclei grown on the impurities often create aggregates, that prevent the movement of arising flakes (Figure 2). On the other hand, a clean surface provides ideal conditions for the growth of single individual domains, which is shown in Figure 4. Moreover, the lower the nucleation rate, the higher the tendency for quasi-monocrystalline assemblance observed. This phenomenon is only possible for growth on a liquid surface, which is atomically smooth and does not block the domains from rotating and moving towards each other allowing the self-alignment into an ordered structure, which can be observed in Figure 4. Figure 5 shows that in the case where the graphene flake nucleated on the solid particles of impurities (visible dark areas in the center of the flake, resulting from the nucleation of successive layers of graphene on the same impurity), those flakes cannot match, hence, they grow on each other. In contrast, Figure 4 shows nucleation without the presence of impurities, where the flakes grow separately. Consequently, such flakes can self-organize and adjust, as evidenced in the measurements of the characteristic angles between them.

**Figure 6:**
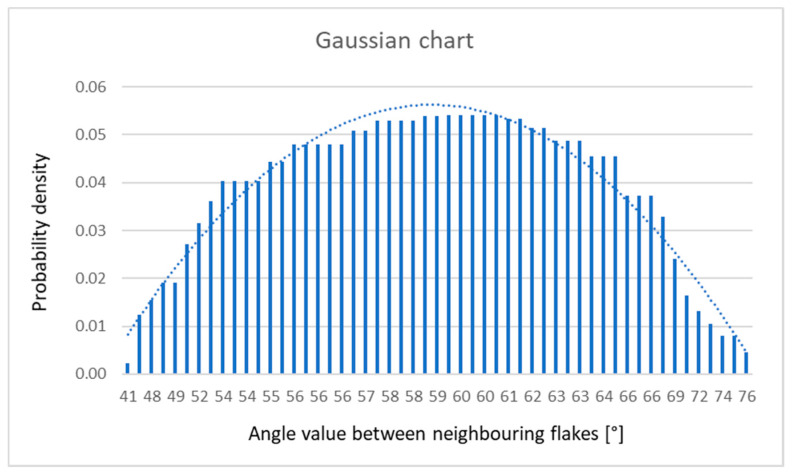
Gaussian chart representing the normal distribution of the angles measured between neighboring flakes.

Figure 4 shows the representative measurement of the misorientation angle. Several other pictures were used for the statistics of the misorientation angle measurement. The statistics are shown in Figure 6, represented by the Gaussian chart. It is clearly visible that most of the appearing graphene flakes with their edge-to-edge matching lead to the formation of low-angle type grain boundaries with a misorientation angle varying between 1° and 4°.

**Figure 7:**
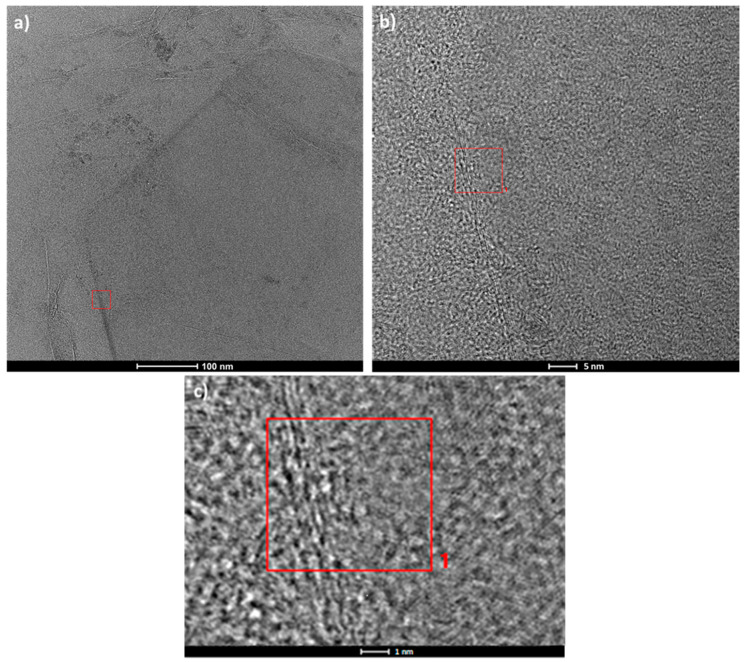
TEM images of (**a**) the graphene flake; (**b**) and (**c**) the magnification of the structure of low-angle grain boundaries.

A transmission electron microscope (TEM) was used for grain boundary examination. Measurement was conducted for graphene grown on a clean surface. This study, as shown in Figure 7, confirmed that in the case of graphene synthesis on a liquid copper substrate, low-angle boundaries occur. Figure 7a–c shows the area of the grain boundaries formed by the growing hexagonal nuclei.

A low-angle boundary is formed on the contact surface of two grains with a misorientation angle smaller than a few degrees. Figure 7 shows that the presence of edge dislocations compensates for the atomic mismatch. Such a boundary is made of a set of one-way edge dislocations, compensating for the mismatch of both parts of the crystal.

**Figure 8:**
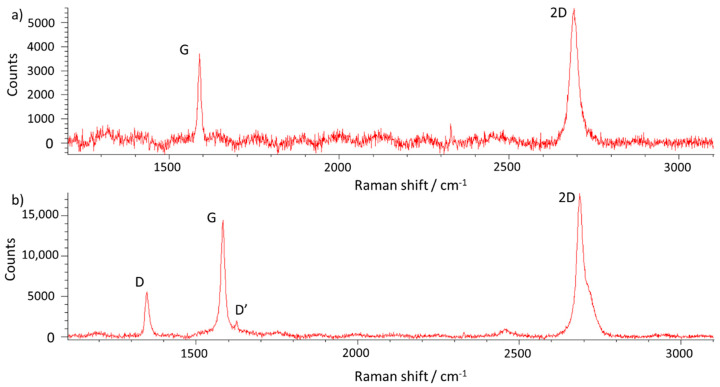
Raman spectrum of (**a**) the graphene layer with the aligned flakes; and (**b**) the graphene layer with locally overlapped flakes and multilayers.

Additionally, Raman spectroscopy was used for the further investigation of the prepared samples. The analysis of the graphene Raman spectra requires three main peaks to take into consideration: D (1350 cm^−1^), G (1580 cm^−1^) and 2D (2690 cm^−1^). Among others, it is possible to evaluate the number of layers and structural defects by the analysis of the peak area, position, intensity and intensity ratios of spectrum peaks. In the spectrum analysis, the relative intensities of individual peak values are important, not the absolute counts’ values. The D peak intensity and I_D_/I_G_ intensities ratio increases with the increase in the number of graphene layer defects [60,61]. Figure 8 shows the Raman spectrum of the prepared samples.

The Raman spectrum of the sample with aligned graphene flakes shown (Figure 8a), is typical for a monolayer graphene with a narrow, symmetric and strong 2D peak. The absence of the D peak suggests a good quality of the graphene structure. The I_2D_/I_G_ ratio is 1.52, proving the monolayer character of the sample. No additional peaks are visible in the spectra. On the other hand, the spectrum of locally overlapped flakes represented in Figure 8b shows substantial structural disorder. The presence of the pronounced D peak, as well as the I_D_/I_G_ ratio equal to 0.37, shows significant defects in the graphene structure. A more defective structure is also supported by the presence of the D′ peak, which results from disorders in the graphene layer and appears around 1620 cm^−1^ [62]. The 2D peak in the Figure 8b Raman spectra is also strong, but its geometry is asymmetrical. The asymmetry of the 2D peak is caused by atomic vibrations in different layers. The interactions between atoms in different layers cause scattering processes, giving rise to other peaks in the 2D spectrum. The consequence of this phenomenon is a wider, asymmetrical 2D peak. [62]. The I_2D_/I_G_ ratio is equal to 1.16, which could even represent a monolayer, but the shape of the 2D peak is more characteristic of a multilayer structure. This can be explained by the local overlapping of the graphene flakes.

## 4. Conclusions

Graphene growth on a liquid metal surface was examined and presented in this paper. The study shows that the obtained graphene grains were of low-angle type due to their ability to move on the surface of the substrate leading to a controlled flakes arrangement. The study also indicates that a lower number of graphene nuclei is favorable for the formation of a monolayer without overlapping zones. The fewer the nuclei, the more possibilities for movement and rotation, as well as a higher probability of perfect alignment. The increasing number of nuclei may result in mutual interference between flakes, which will immobilize them and therefore, prevent them from creating a continuous sheet. The clean and smooth surface of liquid allows graphene flakes to rearrange and lean towards each other creating a quasi-monocrystalline structure. However, when the surface of the growth substrate is contaminated with foreign particles, a perfect alignment is impossible due to the heterogeneous nucleation of graphene on those particles. This leads to a higher nucleation rate, the immobilization of occurring graphene flakes and the growth of additional layers. In order to obtain a monolayer graphene, a fundamental issue is to keep the substrate and process purity at the highest level.

## References

[1] Mukanova A., Tussupbayev R., Sabitov A., Bondarenko I., Nemkaeva R., Aldamzharov B., Bakenov Z. (2017). CVD graphene growth on a surface of liquid gallium. Mater. Today Proc..

[2] Amini S., Garay J., Liu G., Balandin A.A., Abbaschian R. (2010). Growth of large-area graphene films from metal-carbon melts. J. Appl. Phys..

[3] Ding G., Zhu Y., Wang S., Gong Q., Sun L., Wu T., Xie X., Jiang M. (2013). Chemical vapor deposition of graphene on liquid metal catalysts. Carbon.

[4] Tan L., Zeng M., Zhang T., Fu L. (2015). Design of catalytic substrates for uniform graphene films: From solid-metal to liquid-metal. Nanoscale.

[5] Fu Y., Wei Q., Zhang G., Zhong Y., Moghimian N., Tong X., Sun S. (2019). LiFePO₄-Graphene Composites as High-Performance Cathodes for Lithium-Ion Batteries: The Impact of Size and Morphology of Graphene. Materials.

[6] Xu L., Liu S.-K., Lu L.-H., Lu L., Han G. (2019). Preparation and Properties of Graphene/Nickel Composite Coating Based on Textured Surface of Aluminum Alloy. Materials.

[7] Hong X., Fu J., Liu Y., Wang D., Wang X., Dong W., Jiang H. (2019). Recent Progress on Graphene/Polyaniline Composites for High-performance Supercapacitors. Materials.

[8] Warheit D. (2018). Hazard and risk assessment strategies for nanoparticle exposures: How far have we come in the past 10 years?. F1000Research.

[9] Morgeneyer M., Aguerre-Chariol O., Bressot C. (2018). STEM imaging to characterize nanoparticle emissions and help to design nanosafer paints. Chem. Eng. Res. Des..

[10] Zaaba N., Foo K.L., Hashim U., Tan S., Liu W.W., Voon C. (2017). Synthesis of Graphene Oxide using Modified Hummers Method: Solvent Influence. Procedia Eng..

[11] Chen M., Haddon R., Yan R., Bekyarova E. (2017). Advances in transferring chemical vapour deposition graphene: A review. Mater. Horizons.

[12] Guo W., Xu C., Xu K., Deng J., Guo W., Yurgens A., Sun J. (2016). Rapid chemical vapor deposition of graphene on liquid copper. Synth. Met..

[13] Wang J., Zeng M., Tan L., Dai B., Deng Y., Rümmeli M.H., Xu H., Li Z., Wang S., Peng L.-M. (2013). High-mobility graphene on liquid p-block elements by ultra-low-loss CVD growth. Sci. Rep..

[14] Mattevi C., Kim H., Chhowalla M. (2011). A review of chemical vapour deposition of graphene on copper. J. Mater. Chem..

[15] Geng D., Wu B., Guo Y., Huang L., Xue Y., Chen J., Yu G., Jiang L., Hu W., Liu Y. (2012). Uniform hexagonal graphene flakes and films grown on liquid copper surface. Proc. Natl. Acad. Sci. USA.

[16] Chen X., Zhang L., Chen S. (2015). Large area CVD growth of graphene. Synth. Met..

[17] Yin S., Zhang X., Xu C., Wang Y., Wang Y., Li P., Sun H., Wang M., Xia Y., Lin C.-T. (2018). Chemical vapor deposition growth of scalable monolayer polycrystalline graphene films with millimeter-sized domains. Mater. Lett..

[18] Lavin-Lopez M.P., Sanchez-Silva L., Valverde J.L., Romero A. (2017). CVD-graphene growth on different polycrystalline transition metals. AIMS Mater. Sci..

[19] DiMarco C., Robillos T., Hone J., Kysar J. (2018). Mechanisms and criteria for failure in polycrystalline graphene. Int. J. Solids Struct..

[20] Yazyev O.V., Chen Y.P. (2014). Polycrystalline graphene and other two-dimensional materials. Nat. Nanotechnol..

[21] Nguyen V.L., Shin B.G., Duong D.L., Kim S.T., Perello D., Lim Y.J., Yuan Q., Ding F., Jeong H.Y., Shin H.S. (2014). Seamless Stitching of Graphene Domains on Polished Copper (111) Foil. Adv. Mater..

[22] Wang J., Chen L., Wu N., Kong Z., Zeng M., Zhang T., Zhuang L., Fu L. (2016). Uniform graphene on liquid metal by chemical vapour deposition at reduced temperature. Carbon.

[23] Robertson A., Warner J.H. (2011). Hexagonal Single Crystal Domains of Few-Layer Graphene on Copper Foils. Nano Lett..

[24] Wu X., Zhong G., D’Arsie’ L., Sugime H., Esconjauregui S., Robertson A., Robertson J. (2016). Growth of Continuous Monolayer Graphene with Millimeter-sized Domains Using Industrially Safe Conditions. Sci. Rep..

[25] Zhao L., Rim K., Zhou H., He R., Heinz T., Pinczuk A., Flynn G., Pasupathy A. (2011). Influence of copper crystal surface on the CVD growth of large area monolayer graphene. Solid State Commun..

[26] Ding D., Solís-Fernández P., Yunus R.M., Hibino H., Ago H. (2017). Behavior and role of superficial oxygen in Cu for the growth of large single-crystalline graphene. Appl. Surf. Sci..

[27] Chen J., Cui M., Wu G., Wang T., Mbengue J.M., Li Y., Li M. (2017). Fast growth of large single-crystalline graphene assisted by sequential double oxygen passivation. Carbon.

[28] Zeng M., Tan L., Wang L., Mendes R.G., Qin Z., Huang Y., Zhang T., Fang L., Zhang Y., Yue S. (2016). Isotropic Growth of Graphene toward Smoothing Stitching. ACS Nano.

[29] Geng D., Meng L., Chen B., Gao E., Yan W., Yan H., Luo B., Xu J., Wang H., Mao Z. (2014). Controlled Growth of Single-Crystal Twelve-Pointed Graphene Grains on a Liquid Cu Surface. Adv. Mater..

[30] Fujita J.-I., Miyazawa Y., Ueki R., Sasaki M., Saito T. (2010). Fabrication of Large-Area Graphene Using Liquid Gallium and Its Electrical Properties. Jpn. J. Appl. Phys..

[31] Artyukhov V.I., Hao Y., Ruoff R.S., Yakobson B.I. (2015). Breaking of Symmetry in Graphene Growth on Metal Substrates. Phys. Rev. Lett..

[32] Fujita J.-I., Ueki R., Nishijima T., Miyazawa Y. (2011). Characteristics of graphene FET directly transformed from a resist pattern through interfacial graphitization of liquid gallium. Microelectron. Eng..

[33] Yan P., Jeong Y.J., Islam M.F., Pistorius P.C. (2016). Real time and in situ observation of graphene growth on liquid metal surfaces via a carbon segregation method using high-temperature confocal laser scanning microscopy. RSC Adv..

[34] Hiyama T., Murakami K., Kuwajima T., Takeguchi M., Fujita J.-I. (2015). Low-temperature growth of graphene using interfacial catalysis of molten gallium and diluted methane chemical vapor deposition. Appl. Phys. Express.

[35] Wu Y.A., Fan Y., Speller S.C., Creeth G.L., Sadowski J.T., He K., Robertson A., Allen C.S., Warner J.H. (2012). Large Single Crystals of Graphene on Melted Copper Using Chemical Vapor Deposition. ACS Nano.

[36] Zeng M., Tan L., Wang J., Chen L., Rümmeli M.H., Fu L. (2014). Liquid Metal: An Innovative Solution to Uniform Graphene Films. Chem. Mater..

[37] Kula P., Pietrasik R., Dybowski K., Atraszkiewicz R., Szymański W., Kolodziejczyk L., Niedzielski P., Nowak D. (2014). Single and Multilayer Growth of Graphene from the Liquid Phase. Appl. Mech. Mater..

[38] Kula P., Pietrasik R., Dybowski K., Atraszkiewicz R., Kaczmarek L., Kazimierski D., Niedzielski P., Modrzyk W. (2013). The growth of a polycrystalline graphene from a liquid phase. Nanotech, Technical Proceedings of the 2013 NSTI Nanotechnology Conference and Expo, NSTI-Nanotech 2013.

[39] Kula P., Pietrasik R., Kazimierski D., Atraszkiewicz R., Dybowski K., Szymański W., Klimek L., Niedzielski P., Clapa M. (2017). Resistance-temperature characteristics of CVD and high strength metallurgical graphene. Int. J. Nanotechnol..

[40] Kula P., Szymański W., Kolodziejczyk L., Atraszkiewicz R., Dybowski K., Grabarczyk J., Pietrasik R., Niedzielski P., Kaczmarek Ł., Clapa M. (2015). High Strength Metallurgical Graphene—Mechanisms of Growth and Properties/Grafen Metalurgiczny O Wysokiej Wytrzymałości—Mechanizmy Wzrostu I Właściwości. Arch. Met. Mater..

[41] Kula P., Rzepkowski A., Pietrasik R., Atraszkiewicz R., Dybowski K., Modrzyk W. (2016). Method of Producing Graphene from Liquid Metal. U.S. Patent.

[42] Dong J., Wang H., Peng H., Liu Z., Zhang K., Ding F. (2016). Formation mechanism of overlapping grain boundaries in graphene chemical vapor deposition growth. Chem. Sci..

[43] Min S.Y., Cho C., Shim G.W., Park I.-J., Jung D.Y., Woo Y., Lee J.-Y., Choi S.-Y. (2018). Two-dimensional sheet resistance model for polycrystalline graphene with overlapped grain boundaries. FlatChem.

[44] Lee J.-Y., Lee J.-H., Kim M.J., Dash J.K., Lee C.-H., Joshi R., Lee S., Hone J., Soon A., Lee G.-H. (2017). Direct observation of grain boundaries in chemical vapor deposited graphene. Carbon.

[45] Park S., Shehzad M.A., Khan M.F., Nazir G., Eom J., Noh H., Seo Y. (2017). Effect of grain boundaries on electrical properties of polycrystalline graphene. Carbon.

[46] Liu Y., Yakobson B.I. (2010). Cones, Pringles, and Grain Boundary Landscapes in Graphene Topology. Nano Lett..

[47] Nie S., Wofford J.M., Bartelt N.C., Dubon O.D., Mccarty K. (2011). Origin of the mosaicity in graphene grown on Cu(111). Phys. Rev. B.

[48] Biró L.P., Lambin P. (2013). Grain boundaries in graphene grown by chemical vapor deposition. New J. Phys..

[49] Tsen A.W., Brown L., Levendorf M.P., Ghahari F., Huang P.Y., Havener R.W., Ruiz-Vargas C.S., Muller D.A., Kim P., Park J. (2012). Tailoring Electrical Transport Across Grain Boundaries in Polycrystalline Graphene. Science.

[50] Lee W., Kihm K.D., Kim H.G., Shin S., Lee C., Park J.S., Cheon S., Kwon O.M., Lim G., Lee W. (2017). In-Plane Thermal Conductivity of Polycrystalline Chemical Vapor Deposition Graphene with Controlled Grain Sizes. Nano Lett..

[51] Lee D., Kwon G.D., Kim J.H., Moyen E., Lee Y.H., Baik S., Pribat D. (2014). Significant enhancement of the electrical transport properties of graphene films by controlling the surface roughness of Cu foils before and during chemical vapor deposition. Nanoscale.

[52] Incze P.N.-, Vancsó P., Osváth Z., Márk G.I., Jin X., Kim Y.S., Hwang C., Lambin P., Chapelier C., Péterbiró L. (2013). Electronic states of disordered grain boundaries in graphene prepared by chemical vapor deposition. Carbon.

[53] Jhon Y.I., Zhu S.-E., Ahn J.-H., Jhon M.S. (2012). The mechanical responses of tilted and non-tilted grain boundaries in graphene. Carbon.

[54] Grantab R., Shenoy V.B., Ruoff R.S. (2010). Anomalous Strength Characteristics of Tilt Grain Boundaries in Graphene. Science.

[55] Tsen A.W., Brown L., Havener R.W., Park J. (2012). Polycrystallinity and Stacking in CVD Graphene. Accounts Chem. Res..

[56] Akinwande D., Brennan C.J., Bunch J.S., Egberts P., Felts J.R., Gao H., Huang R., Kim J., Li T., Li Y. (2017). A review on mechanics and mechanical properties of 2D materials—Graphene and beyond. Extreme Mech. Lett..

[57] Rao R., Pierce N., Xu Q., Harutyunyan A.R. (2014). Probing inhomogeneous doping in overlapped graphene grain boundaries by Raman spectroscopy. Carbon.

[58] Lee G.-H., Cooper R., An S.J., Lee S., Van Der Zande A.M., Petrone N., Hammerberg A.G., Lee C., Crawford B., Oliver W. (2013). High-Strength Chemical-Vapor-Deposited Graphene and Grain Boundaries. Science.

[59] Zhang X., Xu Z., Yuan Q., Xin J.H., Ding F. (2015). The favourable large misorientation angle grain boundaries in graphene. Nanoscale.

[60] Ferrari A.C. (2007). Raman spectroscopy of graphene and graphite: Disorder, electron–phonon coupling, doping and nonadiabatic effects. Solid State Commun..

[61] Pimenta M.A., Dresselhaus G., Dresselhaus M.S., Cançado L.G., Jorio A., Saito R. (2007). Studying disorder in graphite-based systems by Raman spectroscopy. Phys. Chem. Chem. Phys..

[62] Malard L., Pimenta M., Dresselhaus G., Dresselhaus M. (2009). Raman spectroscopy in graphene. Phys. Rep..

